# Molybdenum titanium carbide (Mo_2_TiC_2_T_*x*_) MXene coated carbon electrodes for vanadium redox flow batteries†

**DOI:** 10.1039/d5ra01163a

**Published:** 2025-04-29

**Authors:** Emil Botling, Ritambhara Gond, Anupma Thakur, Babak Anasori, Amirreza Khataee

**Affiliations:** a Division of Applied Electrochemistry, Department of Chemical Engineering, KTH Royal Institute of Technology SE-100 44 Stockholm Sweden khat@kth.se; b Department of Chemistry – Ångström Laboratory Uppsala University Box 538 751 21 Uppsala Sweden; c School of Materials Engineering, Purdue University West Lafayette IN 47907 USA; d School of Mechanical Engineering, Purdue University West Lafayette IN 47907 USA

## Abstract

Carbon-based electrodes are the most commonly used electrode materials for vanadium redox flow batteries (VRFBs). Due to the use of aqueous electrolytes in VRFBs, the first challenge is the hydrophobicity properties of carbon-based electrodes, and the second challenge is that the desired redox reaction on the positive side, VO^2+^/VO_2_^+^, competes with the oxygen evolution reaction. Therefore, a proper surface treatment is needed. In the present work, three different brands of carbon papers (Sigracet 28AA, Toray 060, and Freudenberg H23) were treated with heat treatment and an MXene coating. For the latter, a two-dimensional (2D) molybdenum titanium carbide (Mo_2_TiC_2_T_*x*_) was chosen and the drop-casting method was used for coating on carbon papers. Scanning electron microscopy (SEM) confirmed MXene distribution and X-ray photoelectron spectroscopy (XPS) showed the presence of Mo and Ti on the electrode surface. Cyclic voltammetry tests revealed that the vanadium reaction rate, 7.76 × 10^−4^ cm s^−1^, and diffusion coefficient, 5.51 × 10^−5^ cm^2^ s^−1^, using Mo_2_TiC_2_T_*x*_ MXene-coated carbon papers are comparable with when heat-treated carbon paper, 1.41 × 10^−3^ cm s^−1^ and 1.32 × 10^−4^ cm^2^ s^−1^, is used. VRFB tests were conducted over 150 cycles. Although a higher resistance of 1.2 Ω cm^2^ was observed for VRFB using Mo_2_TiC_2_T_*x*_ MXene-coated carbon papers *versus* heat-treated, 0.8 Ω cm^2^, the energy efficiency of 71% was reasonably comparable to 79% for the system using heat-treated electrodes. More importantly, the same discharge capacity retention of 75% was achieved for both systems. The chemical stability of the Mo_2_TiC_2_T_*x*_ MXene coating was confirmed by XPS post-analysis of electrodes where similar peaks for the freshly coated electrodes were observed. This work further broadens the potential applications of MXene coating as a treatment for carbon electrodes.

## Abstract 2

Carbon-based electrodes are the most commonly used electrode materials for vanadium redox flow batteries (VRFBs).

## Graphical abstract

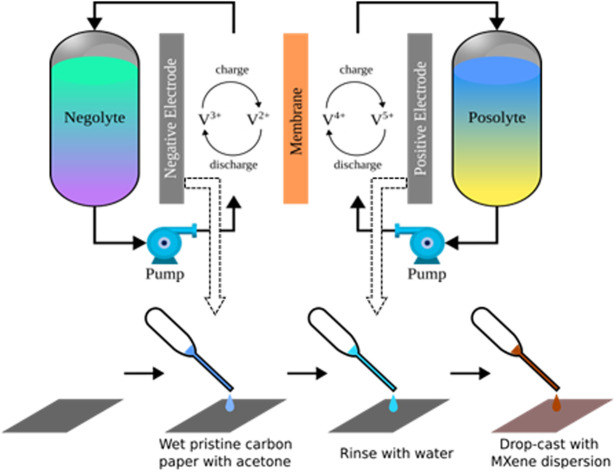

## Introduction

The flexibility of redox flow batteries (RBFs) in terms of configuration and operation offers excellent advantages for stationary energy storage. This is due to the independently scalable power and capacity, in contrast to conventional batteries (lithium-ion batteries, for example), where power and capacity depend on each other. Other advantages include long-duration discharge time, long lifespan, and good stability.^1^ The most mature and commercially available RBF is based on vanadium chemistry using four different oxidation states: V^2+^/V^3+^ at the negative side and VO^2+^/VO_2_^+^ at the positive side.^2^ Using the same elements on both sides avoids the cross-contamination of the electrolytes. The aqueous vanadium electrolytes are non-flammable and non-explosive, and no toxic gases are generated by cycling.^3^ Vanadium redox flow batteries (VRFBs) do not suffer from high depth of discharge and offer a long life span (+20 years). One of the critical components that plays a significant role in the cost and operation of VRFBs is the electrode. Electrodes, where the electrochemical reactions occur, must provide surface area, catalytic effect, hydrophilicity, conductivity, and stability.^4^ Carbon-based electrodes (*e.g.*, carbon paper) are the most common materials used in VRFBs.^5–7^ The proportion of carbon-based electrode costs to total system costs is estimated to be below 5%.^8^ Low weight, high porosity (>80%), high conductivity (>450 mS cm^−1^), and good stability in aqueous media are the main properties of carbon-based electrodes; however, their active surface area, catalytic properties, and hydrophilicity highly depend on the treatment type.^4–7,9^ The most common method is heat treatment, which solves the mentioned challenges to a certain extent by introducing oxygen functional groups to the electrode surface.^9^ The significant outcome is a hydrophilic surface with active sites for electrochemical reactions. Another strategy is to coat the carbon-based electrodes with metal and metal oxide electrocatalysts to improve the catalytic properties and increase conductivity.^10^ However, preparing coating ink using particular ionomers in organic solvents is complicated and could be costly due to using platinum-group metals. In addition, the electrodes do not become hydrophilic. Here, one can think about gaining the benefits of both methods using a single process of MXene coating on the electrodes.

MXenes are a family of two-dimensional (2D) transition metal carbides, nitrides, and carbonitrides.^11^ MXenes have characteristics such as hydrophilicity and negative zeta potential in the range of −30 mV to −50 mV at pH 7, high metallic electrical conductivity (∼24 000 S cm^−1^ for Ti_3_C_2_T_*x*_ MXene), and high mechanical strength.^11–13^ The general formula of MXenes is M_*n*+1_X_*n*_T_*x*_ (*n* = 1, 2, 3…), where M represents early transition metals (Ti, Mo, Cr, Nb, *etc.*), X represents carbon or nitrogen while T_*x*_ represents surface functional groups (–OH, –O, –F, *etc.*).^14^ There are over 60 MXenes with different compositions, and due to the above-mentioned beneficial properties, the application of MXenes in the electrochemistry field has been expanding.^15^ MXene materials can be combined with different substrates such as polymers, oxides, and carbon nanotubes (CNTs) to obtain MXene hybrids.^11^ This is due to the high flexibility, 2D morphologies, and layered architectures.^16,17^ Many substrate materials have been combined with MXenes to form MXene hybrid materials. These secondary materials include transition metal compounds (oxides, phosphides, chalcogenides, *etc.*), layered double hydroxides (LDH), carbon-based (graphene, CNTs, *etc.*), metals and metal alloys as well as supramolecular structures like metal–organic frameworks (MOFs).^11^

Recently, a few promising attempts have shown the application of MXenes in VRFBs.^18^ The metal-like electrical conductivity, large surface area, and hydrophilicity of MXenes enable them to be used on the electrodes. MXene-coated carbon electrodes have shown good capability in catalyzing V^2+^/V^3+^ electrochemical reactions on the negative side of VRFBs while inhibiting the hydrogen evolution reaction (HER).^19,20^ In our previous work,^4^ we developed a method to coat Ti_3_C_2_T_*x*_ MXene on carbon papers for VRFBs. The VRFB using the MXene-coated electrode on the negative side showed stable discharge capacity for over 100 cycles. In addition, competitive energy efficiency (∼70%) was achieved at 130 mA cm^−2^ compared to VRFB using heat-treated electrodes. Despite the negative side reaction (V^2+^/V^3+^), the Ti_3_C_2_T_*x*_ MXene-coated carbon papers did not show superior performance *versus* heat-treated samples for positive side reaction (VO^2+^/VO_2_^+^). Therefore, this work investigates the electrochemical performance of carbon papers coated with double-transition metal (DTM), molybdenum titanium carbide (Mo_2_TiC_2_T_*x*_) MXene for the positive side (VO^2+^/VO_2_^+^) of VRFBs. We selected DTM MXene with a similar structure to M_3_C_2_ MXene, with Ti atomic layer sandwiched between two outer Mo atomic layers^21–23^ to understand the effect of Mo on the VO^2+^/VO_2_^+^*versus* oxygen evolution reaction (OER). The main goal is to see how the Mo_2_TiC_2_T_*x*_ MXene-coated electrode catalyzes the VO^2+^/VO_2_^+^ electrochemical reaction *versus* oxygen evolution.

## Experimental

### Materials

Carbon papers (Sigracet 28 AA, Toray 060, and Freudenberg H23) and membrane (Nafion 212) were purchased from FuelCellStore. Vanadyl sulfate hydrate (VOSO_4_), sulfuric acid (H_2_SO_4_), and acetone were purchased from Sigma-Aldrich. Hydrofluoric acid (HF, 48–51% solution in water) was purchased from Acros Organics. Lithium chloride (LiCl, 98% grade, Thermo Scientific) and hydrochloric acid (HCl, 12 M) were purchased from Fisher Scientific and used as received. A commercial 1.6 M vanadium electrolyte (mixture of V^3+^ and VO^2+^) in 2 M H_2_SO_4_ was purchased from the GfE company (Gesellschaft für Elektrometallurgie mbH). All chemicals used in electrochemical tests were used without further purification.

### Heat treatment

The carbon papers (Sigracet 28AA, Toray 060, and Freudenberg H23) were initially heat-treated at 500 °C for 3 hours with a heating rate of 167 °C h^−1^ using a muffle furnace (Nabertherm). The electrochemical performance results of heat-treated electrodes were used as a reference to be compared with MXene-coated electrodes. Also, the most suitable carbon paper was selected for MXnene coating by testing heat-treated samples.

### MXene synthesis

To synthesize Mo_2_TiC_2_T_*x*_ MXene, 1 g of Mo_2_TiAlC_2_ MAX phase was mixed with 10 mL of hydrofluoric acid (HF, 49–51 wt% concentration, Fisher Scientific) solution as an etchant in a high-density polyethylene bottle and stirred at 300 rpm for 96 h at 55 °C. The etched multilayered Mo_2_TiC_2_T_*x*_ MXene flakes were washed with deionized water through repeated centrifugation at 3234 RCF (4–5 cycles with ∼300 mL of deionized water) until the supernatant reached pH ∼ 7. To delaminate, the etched multilayer Mo_2_TiC_2_T_*x*_ MXene sediment was added to 5 mL of tetramethylammonium hydroxide (TMAOH) solution (25 wt% stock, Fisher Scientific) in 20 mL of deionized water per gram of starting Mo_2_TiAlC_2_ MAX. The mixture of TMAOH and etched multilayered Mo_2_TiC_2_T_*x*_ MXene was then stirred at 300 rpm for 4 h at 55 °C. After delamination, the TMA^+^ intercalated Mo_2_TiC_2_T_*x*_ MXene solution was washed to neutral pH *via* repeated centrifugation at 21 913 RCF (4 cycles with ∼300 mL of deionized water). Thereafter, the final mixture of Mo_2_TiC_2_T_*x*_ MXene was re-dispersed in 20 mL of deionized water and vortexed for 15 minutes. The suspension was centrifuged at 3000 RCF for 30 minutes to collect single-to-few layered Mo_2_TiC_2_T_*x*_ MXene flakes. The final Mo_2_TiC_2_T_*x*_ MXene suspension was collected and stored in a freezer at −20 °C until use.

### MXene coating on carbon papers

The concentrated Mo_2_TiC_2_T_*x*_ MXene dispersion was stored in the freezer at −15 °C and cooled to room temperature before coating. Mo_2_TiC_2_T_*x*_ MXene of 5 mg mL^−1^ concentration was sonicated using an ultrasonicator (Vibra Cell, Sonics – Sonics & Materials, Inc.) five times (each for three seconds) with 10 seconds of rest time in between.

The pristine carbon papers, initially possessing hydrophobic properties, were coated with Mo_2_TiC_2_T_*x*_ MXene using a sequential process as in the previous work.^4^ The first step was immersing the electrode in acetone to ensure complete wetting. Following the wetting step, the electrode was rinsed with ultrapure water to remove the acetone from the carbon paper. The water-wetted carbon paper was immediately coated with a 5 mg per mL Mo_2_TiC_2_T_*x*_ MXene dispersion using a micropipette under normal atmospheric conditions. The wet Mo_2_TiC_2_T_*x*_ MXene-coated electrode is dried at 100 °C for 15 minutes using a vacuum oven. Both sides of the carbon paper are coated with 0.1 mL, 0.5 mL, and 1 mL of Mo_2_TiC_2_T_*x*_ MXene dispersion, resulting in coating densities of 0.1 mg cm^−2^, 0.5 mg cm^−2^ and 1.0 mg cm^−2^ on each side and are labeled MX-0.1, MX-0.5, and MX-1, respectively.

### Material characterization

The surface morphology of the electrodes was analyzed using a Hitachi S-4800 SEM with an accelerating voltage of 10 kV, a working distance of 9400 μm, and an emission current of 10.1 μA without sputtering. The surface composition is analyzed with Kratos AXIS Supra+ instrument X-ray photoelectron spectroscopy (XPS) using monochromatic Al Kα radiation (1486.6 eV) with calibration using carbon 1s at 284.5 eV. All XPS data was processed using CasaXPS.

### Cyclic voltammetry

Cyclic voltammetry tests were performed using a three-electrode setup using a platinum wire counter electrode, Ag/AgCl reference electrode, and carbon paper as the working electrode. A VersaSTAT 4 potentiostat was used to run the tests. The electrolyte consisted of 50 mM vanadyl sulfate hydrate (VO^2+^) and 50 mM H_2_SO_4_. The electrode samples were cut into 5 cm^2^ pieces and attached to the working electrode holder. The electrode was submerged until the holder was just above the surface of the electrolyte solution, resulting in a submerged area of 4 cm^2^ of the electrode. The screening process to select the most suitable substrate was performed between 0 and 1.2 V (positive side reaction VO^2+^/VO_2_^+^) and at scan rates of 3, 5, 10, 20, 30, and 50 mV s^−1^. The same voltage range and scan rates were used for the MXene-coated samples.

Moreover, the electrochemical reaction rate and diffusion coefficient were calculated through cyclic voltammetry tests. Through the relationship between peak current density and scan rate, the diffusion coefficient (*D*) was obtained by eqn (1). In addition, the relationship between the peak current density and formal potential was used to obtain the electrochemical reaction rate constant (*k*^0^) by eqn (2).^4,24,25^

1 *j*_p_ = (2.99 × 10^5^)*α*^1/2^*C*^0^*D*^1/2^*v*^1/2^

2
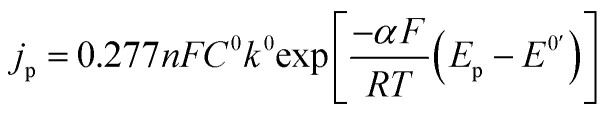
In the equations, *j*_p_ is peak current density based on the geometrical area, *n* number of exchanged electrons, *C*^0^ bulk concentration of the redox species, *D* diffusion coefficient (cm^2^ s^−1^), *ν* scan rate (V s^−1^), *F* Faradays constant (C mol^−1^), *k*^0^ rate of electrochemical reaction constant (cm s^−1^), *α* charge transfer coefficient with the assumption of 0.5, *E*_p_ peak potential, and *E*^0^′ (V) formal potential. The diffusion coefficient can be determined from the slope when the peak current density is plotted against the scan rate (eqn (1)). Moreover, the reaction rate constant can be determined from the slope when the peak current density is plotted against the difference between the peak and formal potential, assuming a value of *α* = 0.5.

The electrochemical active surface area (ECSA) was estimated through cyclic voltammetry of the blank solution, 2 M H_2_SO_4_, within non-faradic region shown in Fig. S2.†

Considering the voltage difference (Δ*V*) between 0.95 V and 1.05 V at different scan rates, the double-layer capacitance was calculated using eqn (3).^26^3
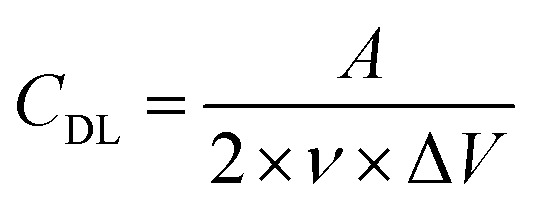
where *C*_dl_ corresponds to double-layer capacitance, and *A* is the area under the cyclic voltammetry curve within Δ*V*. Using the obtained double-layer capacitance (*C*_DL_) and by knowing that the specific capacitance (*C*_S_) of the material, 0.021 F cm^−2^,^27^ the ESCA is calculated through eqn (4).^26^4
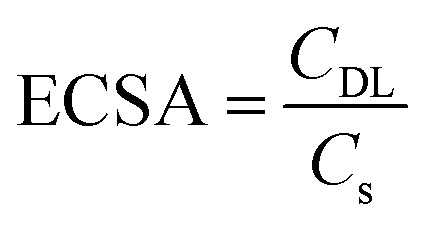


### VRFB tests

VRFB performance was investigated through charge/discharge tests. The setup consists of a cell and electrolyte tanks. An electrolyte containing 1.6 M vanadium (mixture of V^3+^ and VO^2+^) in 2 M H_2_SO_4_ was used. The cell consisted of a Nafion 212 proton exchange membrane, two layers of carbon papers with a geometrical area of 5 cm^2^ placed on graphite plates with a serpentine flow field design. The cell was sealed with Viton gaskets. The total amount of electrolyte was 13 mL on each side, and the negative side was purged with nitrogen gas using an external glass container around the electrolyte container to prevent oxidation of V^2+^. Furthermore, the electrolyte was pumped at a constant volumetric rate of 40 mL min^−1^ using a dual-channel peristaltic pump (BT600L, Zhengzhou Mingyi Instrument Equipment Co., Ltd).

Regarding the test equipment, the CT2001A (Landt Instruments) and a Bio-Logic VSP-300 potentiostat were used for the battery charge and discharge cycling tests. The cycling was performed using constant current protocol at 100 mA cm^−2^ with cut-off voltages of 1.7 V and 0.8 V. The first cycle was excluded from the battery results when evaluating due to the much longer charge/discharge time compared to other cycles.

Electrochemical impedance spectroscopy (EIS) was conducted between 100 kHz and 1 Hz at constant voltage and was performed using the VRFB setup and Bio-Logic VSP-300 potentiostat. The cell was charged and discharged for three cycles and then charged until 1.3 V in open circuit voltage at ∼50% state of charge (SoC) followed by the potentiostatic EIS measurement. From the Nyquist plot of the measured EIS, the charge transfer resistance can be determined from the diameter of the semicircles, as well as the ohmic resistance of the cell from the intersect of the real and imaginary axis.^28^

## Results and discussion

**Fig. 1:**
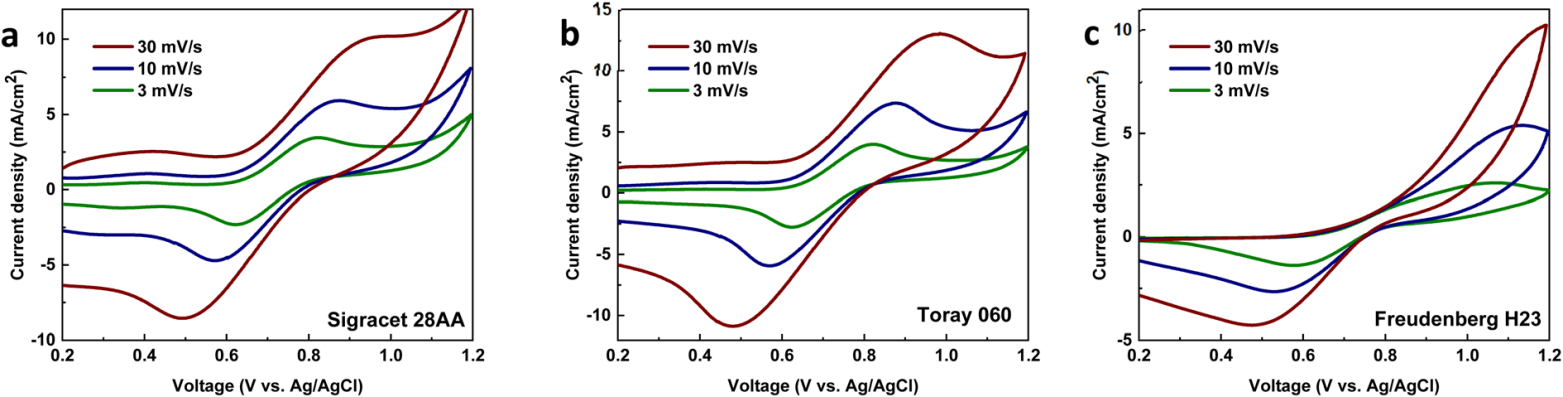
(a–c) Cyclic voltammograms of vanadyl sulfate hydrate (VO^2+^/VO_2_^+^) on three different heat-treated carbon papers. Measured between 0.2–1.2 V *vs.* Ag/AgCl at different scan rates. The electrolyte consists of 50 mM vanadyl sulfate (VO^2+^) and 50 mM H_2_SO_4_.

The electrochemical evaluation of electrodes was conducted first for the heat-treated carbon papers through cyclic voltammetry of VO^2+^/VO_2_^+^ (Fig. 1). The main goal is to select the best heat-treated carbon paper out of three (Sigracet 28AA, Toray 060, and Freudenberg H23) and later compare it with MXene-coated carbon papers. The oxidation and reduction peaks are visible in all scan rates using Sigracet 28AA and Toray 060, showing the catalytic properties of the carbon papers for the primary electrochemical reaction (VO^2+^/VO_2_^+^) *versus* oxygen evolution. However, using Freudenberg H23, while the reduction peaks are visible at different scan rates, the oxidation peak becomes invisible when the scan rate is increased. The latter is more likely because the electrode has more selectivity towards oxygen evolution than the primary electrochemical reaction.

For further analysis, the peak current ratio and peak separation voltage were calculated for each test, and the results are shown in Fig. S1a and S1b.† It was assumed that the ideal reversible electrochemical reaction shows a peak current ratio of 1 and a peak separation voltage of 59 mV in cyclic voltammetry tests.^29,30^ It can be seen that the peak current ratio using Sigracet 28AA and Toray 060, with a similar trend, is the closest to the ideal value, while using Freudenberg H23, it shows deviation. The peak separation voltage for all carbon papers deviates from the ideal value (>200 mV), meaning that the electrochemical reaction of vanadium is quasi-reversible on the electrodes. Still, Sigracet 28AA showed the slightest increase in peak separation voltage by increasing the scan rate. Moreover, the cyclic voltammograms were analyzed to obtain the vanadium diffusion coefficient and rate of electrochemical reaction using Fig. S1c, S1d† and eqn (1)–(3). The results can be seen in Table 1. The vanadium diffusion coefficient is higher on Sigracet 28AA and Toray 060 compared to Freudenberg H23. Still, using Toray 060, the diffusion coefficient is highest. We correlate the data in Table 1 to the difference in the specification of carbon papers, mainly porosity and resistivity.

**Table 1:** Vanadium diffusion coefficients (*D*), electrochemical reaction rate (*k*^0^) and electrochemical surface area (ECSA) on different carbon papers. All data were extracted from cyclic voltammetry tests. All carbon papers were heat-treated

Substrates	*D* (cm^2^ s^−1^)	*k* ^0^ (cm^2^ s^−1^)	ECSA (cm^2^)
Sigracet 28AA	3.80 × 10^−5^	1.31 × 10^−3^	34.49
Toray 060	6.83 × 10^−5^	2.40 × 10^−3^	38.47
Freudenberg H23	3.73 × 10^−5^	1.45 × 10^−3^	46.10

The vanadium reaction on Freudenberg H23 showed the most sluggish rate constant, while the Toray 060 performed the best catalytic effect with the highest value for the rate of reaction constant at 2.40 × 10^−3^ cm s^−1^. Considering the mentioned evaluations, between the Sigracet 28AA and Toray 060, the Sigracet 28AA showed better performance in terms of oxidation and reduction peaks, reversibility, and selectivity. In comparison, the Toray 060 showed better performance concerning the diffusion coefficient and rate of electrochemical reaction.

The ECSA was estimated for the different carbon papers and determined by the double-layer capacitance estimation. The resulting measurements for the double-layer capacitance can be seen in the ESI in Fig. S2a–c,† and the linear correlation between the logarithm of the current (both anodic and cathodic) at 1 V *versus* the logarithm of the scan rate would provide double-layer capacitance extrapolated from the slope of the linear correlation. By knowing the estimated specific capacitance of carbon papers, the ECSA of samples was calculated using eqn (4). Table 1 shows the ECSA values where Freudenberg H23 has the highest.

As the higher surface area of an electrode generally improves the output power of the battery, the Freudenberg H23 seems like the most optimal choice. However, it performed poorly compared to the Sigracet and Toray samples in the cyclic voltammetry measurements of the vanadium electrolyte (Fig. 1c). The Freudenberg H23 electrode seems to have a higher selectivity towards the OER than the Sigracet 28AA and Toray 060 samples. Furthermore, from the results of the estimation of the ECSA, the Toray 060 showed a higher ECSA compared to the Sigracet 28AA sample. This results in higher performance for the Toray 060 compared with Sigracet 28AA, as a higher electrode surface area is more desirable when optimizing for higher power while keeping the catalytic effect of the electrode surfaces.

Although ECSA is an essential metric of the performance and characteristics of an electrode, the catalytic effect and performance for the desired redox species are also significant. Hence, the Freudenberg H23 samples were not compared further with the Toray 060 and Sigracet 28AA samples.

**Fig. 2:**
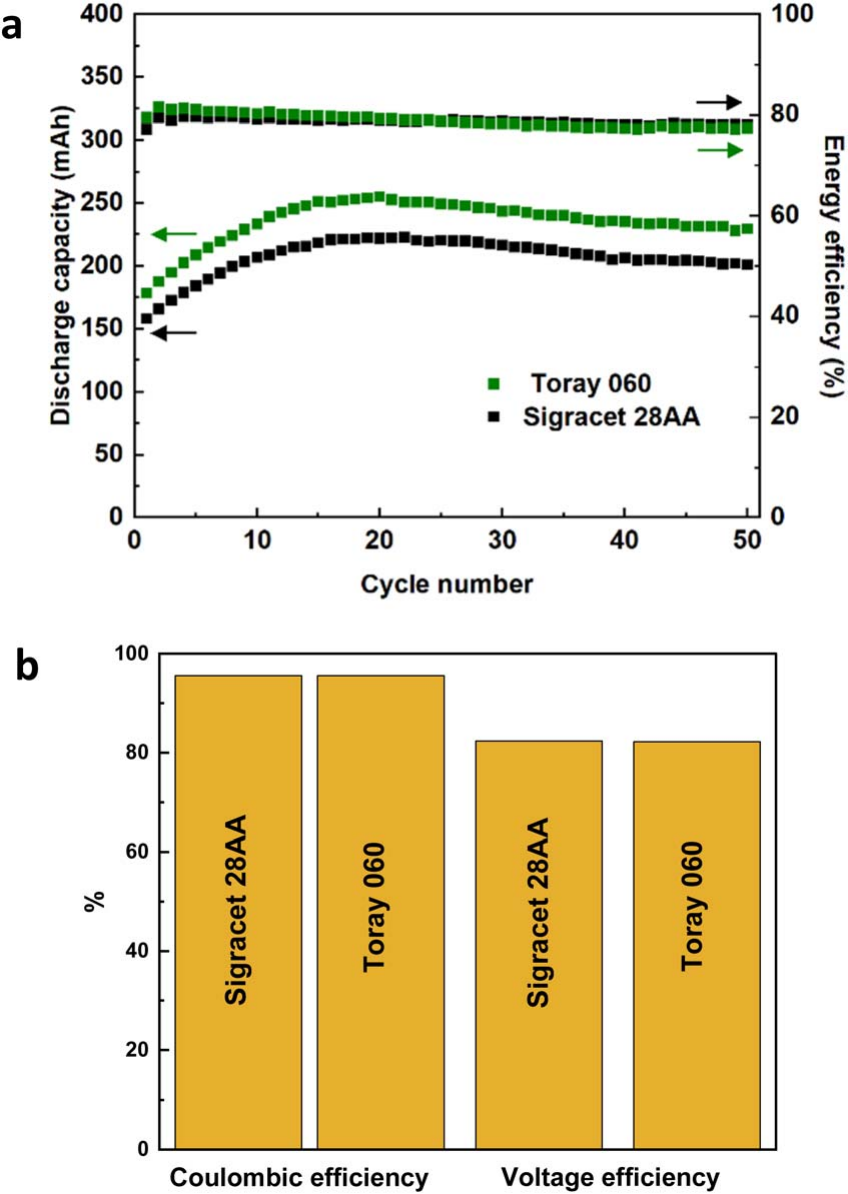
VRFB performance using heat-treated Sigracet 28AA and Toray 060 carbon papers; (a) discharge capacity and energy efficiency, (b) coulombic and voltage efficiencies. The VRFB tests were conducted at a flow rate of 40 mL min^−1^ with 13 mL of the electrolyte on each side and was charged/discharged at constant current 100 mA cm^−2^ between 0.8 and 1.7 V. Two layers of carbon papers were stacked on each side of the cell.

To further compare the electrochemical performance of heat-treated carbon papers, VRFB tests were conducted using Sigracet 28AA and Toray 060. The results of 50 charge/discharge cycles can be seen in Fig. 2. The VRFBs using samples exhibit a peak in discharge capacity at around 20 cycles, which then decreases until a plateau is reached. This behavior is connected to severe non-equilibrium water crossover using Nafion membrane as was reported in our previous studies.^3,31,32^ The capacity retention for the VRFB using Toray 060 was better compared to using Sigracet 28AA as the values at cycle number 50 were around 240 mA h and 210 mA h, respectively. However, in terms of coulombic, voltage, and energy efficiencies, the VRFBs using both carbon papers exhibited identical results. A little bit more detailed analysis, the energy efficiency is slightly lower in initial cycles in the case of Sigracet 28AA compared to the Toray 060 but the average energy efficiency is slightly higher and more stable during 50 cycles for the VRFB using Sigracet 28AA. Overall, from the electrochemical data and analysis, both Sigracet 28AA and Toray 060 carbon papers are suitable reference samples to compare with MXene-coated samples. However, as Fig. S3† shows, when attempting to coat the carbon paper following the protocol mentioned earlier in electrode preparation, the untreated Toray sample was not successfully wetted as the MXene ink was not evenly coated on the surface. Therefore, Sigracet 28AA was chosen as the suitable substrate for the MXene coating, and the electrochemical performance of MXene-coated samples will later be compared with heat-treated Sigracet 28AA.

**Fig. 3:**
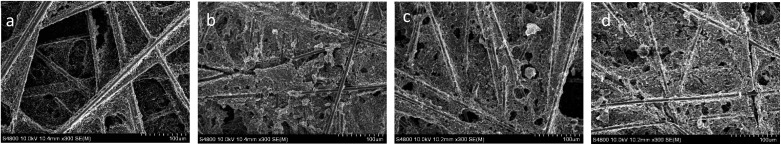
SEM images: (a) heat-treated Sigracet 28AA; untreated Sigracet 28AA coated with MXene ink with a coating density of (b) 0.1 mg cm^−2^, (c) 0.5 mg cm^−2^ and (d) 1 mg cm^−2^.

For MXene coating, untreated Sigracet 28AA was coated with Mo_2_TiC_2_T_*x*_ MXene at different surface densities (0.1, 0.5, and 1 mg cm^−2^) and their SEM images were compared with the heat-treated Sigracet 28AA. Fig. 3 shows the structure difference between samples based on their treatment type.

Comparing Fig. 3a–d, it is shown that MXene has successfully been coated on carbon paper as the pores between the carbon fibers are more or less filled depending on the surface loading condition. Overall, the MXene seems relatively evenly distributed on the surface of the carbon paper electrodes. Although the lowest coating density, the Sigracet 28AA coated with 0.1 mg cm^−2^ (MX-0.1) showed more uniform coating while coated samples with 0.5 and 1 mg cm^−2^ (MX-0.5 and MX-1) show similar coated surface with some visible pores which could potentially increase the hydraulic permeability.^33^ This could be due to fewer layers of Mo_2_TiC_2_T_*x*_ in lower coating densities that allow a synergistic effect of carbon and MXene.

**Fig. 4:**
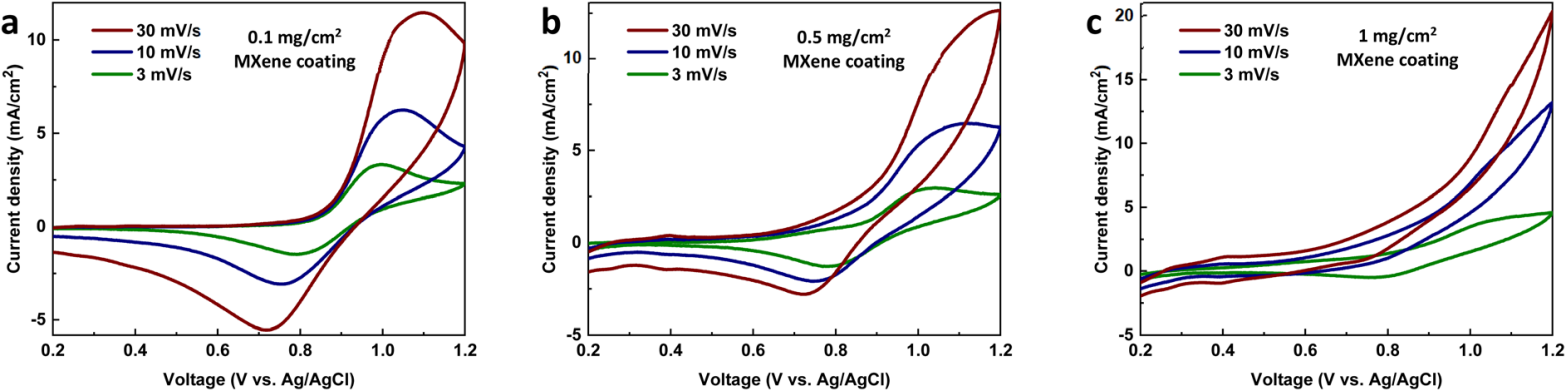
Cyclic voltammograms of coated Sigracet 28AA with different coating densities of Mo_2_TiC_2_T_*x*_ MXene; (a) 0.1 mg cm^−2^ (MX-0.1), (b) 0.5 mg cm^−2^ (MX-0.5) and (c) 1 mg cm^−2^ (MX-1). The electrolyte consists of 50 mM vanadyl sulfate (VO^2+^) and 2 M sulfuric acid.

Following SEM analysis, cyclic voltammetry tests were conducted for Mo_2_TiC_2_T_*x*_ MXene-coated Sigracet 28AA samples (Fig. 4). A higher concentration of H_2_SO_4_ (2 M) was used compared to previous tests to avoid any risk of undistinguishable peaks and to increase conductivity.^34^ It should be noted that the heat-treated Sigracet 28AA showed identical electrochemical performance compared to the electrolyte with a lower acid concentration (50 mM), and we do not replicate it in Fig. 4.

For the Mo_2_TiC_2_T_*x*_ MXene-coated samples, a trend can be seen in Fig. 4a–c where the oxidation/reduction peaks for the VO^2+^/VO_2_^+^ reaction are sharper using MX-0.1, less sharp using MX-0.5 and not distinguishable using MX-1 sample indicating that the lowest coating density shows the best performance. This is coherent with the SEM analysis, where MX-0.1 likely has better MXene coating than MX-0.5 and MX-1.

The diffusion coefficients and electrochemical reaction rate constants were calculated for MXene-coated samples through cyclic voltammetry measurements. It should be noted that the peaks are not sharp enough for MX-0.5 and MX-1 samples, so the values were not calculated as the data would give inaccurate values for the coefficients. The diffusion coefficient and electrochemical reaction rate constant for MX-0.1 are 5.51 × 10^−5^ cm^2^ s^−1^ and 7.76 × 10^−4^ cm s^−1^, respectively.

Comparing these data with heat-treated Sigracet 28AA in Table 1, although the heat-treated carbon paper exhibited better kinetic data, the data for MX-0.1 are still competitive. The MXene-coated MX-0.1 sample successfully catalyzed the primary vanadium (VO^2+^/VO_2_^+^) electrochemical reaction *versus* the OER reaction.

More importantly, the kinetic data of MX-0.1 calculated using Fig. S4† are superior to those in our previous work (diffusion coefficient: 4.05 × 10^−6^ cm^2^ s^−1^, electrochemical reaction rate: 4.17 × 10^−4^ cm s^−1^),^4^ where carbon papers were coated with mono transition metal Ti_3_C_2_T_*x*_ MXene and a coating density of 0.1 mg cm^−2^. Furthermore, the peak separation voltage in cyclic voltammetry tests of MX-0.1 in the previous work was 600 mV while it is around 200 mV in this work. These results show that the double transition metal MXene improves the electrochemical performance of carbon papers for the VO^2+^/VO_2_^+^ reaction.

Since the Mo_2_TiC_2_T_*x*_ MXene-coated sample with the lowest coating density (0.1 mg cm^−2^) among other surface densities showed the best performance in the cyclic voltammetry tests, it was selected for VRFB tests. A possible explanation for not using high coating density MXene-coated samples could be that increasing coating density results in the loss of catalytically active sites as the MXene layers make a barrier on the carbon paper. The same trend was observed in our previous work, where the samples with higher coating density than 0.1 mg cm^−2^ exhibited worse performance, and we attributed this trend to the MXene flakes or particles accumulated on carbon paper pores.^4^

**Fig. 5:**
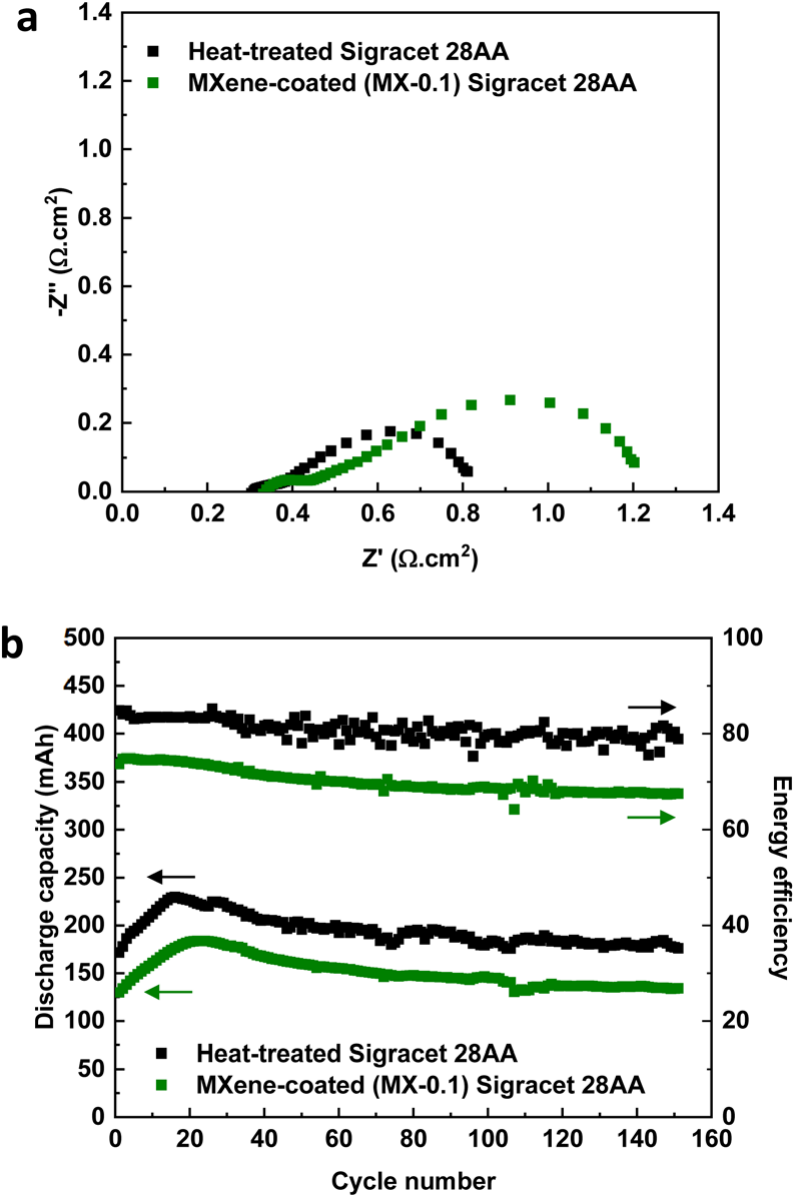
VRFB performance comparison using heat-treated and Mo_2_TiC_2_T_*x*_ MXene coated Sigracet 28AA on the positive side (heat-treated Sigracet 28AA was used on the negative side for both configurations); (a) full cell EIS analysis for, (b) discharge capacity and energy efficiency for 150 cycles of charge/discharge. The VRFB tests were conducted at a flow rate of 40 mL min^−1^ with 13 mL of the electrolyte on each side and were charged/discharged at a constant current of 100 mA cm^−2^ between 0.8–1.7 V. Two layers of electrodes were stacked on each side for the configurations.

Fig. 5 shows the VRFB performance using MX-0.1 on the positive side and heat-treated carbon papers on the negative side. The results are compared with the VRFB using heat-treated carbon papers on both sides. Firstly, the resistance of the VRFB was investigated *via* EIS analysis.

From the Nyquist plot in Fig. 5a, it is shown that when using MX-0.1 on the positive side of VRFB, the total resistance is 0.240 Ω while using heat-treated carbon papers on both sides, a total resistance of 0.162 Ω was achieved. The ohmic resistance is similar for both configurations (0.068 Ω *versus* 0.062 Ω). Therefore, the difference in total resistance could mainly be due to the difference in charge transfer resistance of the vanadium reaction. The first semicircle corresponds to the negative side as it has similar values for both configurations. However, the second semicircle corresponds to the positive side of the VRFB. This is a reasonable conclusion because heat-treated carbon papers are used on the negative side of both configurations while the positive side is different. The higher charge transfer resistance for the VRFB using MX-0.1 means that MXene-coated carbon papers contribute more resistance than heat-treated ones. The VRFB tests were conducted using both configurations. The discharge capacity has a similar trend, as shown in Fig. 2, with a peak in initial cycles connected to how the Nafion membrane works.^3,31,32^ The discharge capacity becomes stable after the peak point (>cycle 20th). Interestingly, VRFB using both configurations shows similar capacity retention. The capacity loss is negligible for both configurations, meaning that capacity loss is not affected by using MXene-coated electrodes. This is a good sign of the stability of MXene-based electrodes. The deviation from the theoretical capacity (∼278 mA h) is due to running the system at a relatively high current density (100 mA cm^−2^). Another important thing is the difference between the discharge capacity of the two configurations. Due to the higher total resistance of VRFB using MX-0.1, the discharge capacity is lower.

The energy efficiency of the VRFB using both configurations showed a stable trend with a slight decrease for over 150 cycles. The lower energy efficiency of MXene-based VRFB is due to the lower voltage efficiency due to higher resistance (Fig. 5a). Fig. S5† provides more details on the efficiencies of the VRFB system. The VRFB using MX-0.1 presented a higher coulombic efficiency than the configuration with only heat-treated carbon paper electrodes. This is an indication of likely fewer side reactions (such as OER) and a lower electrolyte crossover. As mentioned, the VRFB using heat-treated carbon paper electrodes outperformed the system using MX-0.1 electrodes in voltage efficiency (86.62% *versus* 71.7%).

Overall, the VRFB using MXene-coated carbon papers on the positive side showed successful and competitive performance compared to heat-treated electrodes, meaning that MXene coating is an alternative pre-treatment for carbon paper electrodes. Furthermore, the heat-treatment method consumes a lot of energy because of the use of elevated temperature (>500 °C) for several hours (3–12 hours). However, MXene coating is performed *via* a straightforward drop casting protocol and using low coating densities (<0.5 mg cm^−2^).

**Fig. 6:**
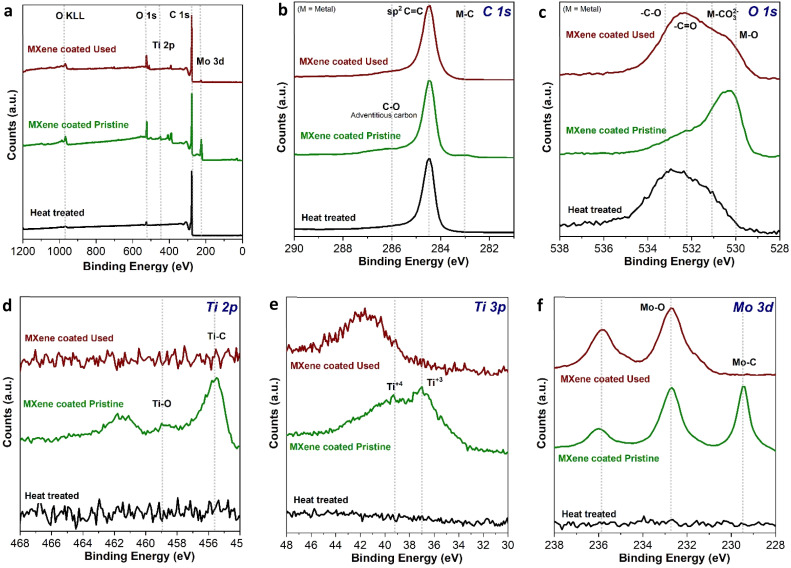
Component XPS peak fits for prepared MXene-coated electrodes (MX-0.1) before and after VRFB tests; (a) wide XPS spectra, (b) carbon 1s, (c) oxygen 1s, (d) titanium 2p, (e) titanium 3p, (f) molybdenum 3d.

The wide spectrum from the XPS analysis of three different samples clearly shows the presence of Ti 2p, Mo 3d, C 1s, and O 1s elemental species, as expected, showing the purity of the sample with no contamination, see Fig. 6a. The C 1s as shown in Fig. 6b, clearly depicts the intrinsic sp^2^ nature of C paper, however a little hump at 283 eV in the MXene-coated pristine C paper revealed the presence of metal carbide (M–C) nature that is arising due to a coating of Mo_2_TiC_2_T_*x*_ hybrid MXene, which still be seen after cycling labeled as MXene coated used in the XPS spectrum. Further based on O 1s spectra from Fig. 6c, spectra of heat treated, Mo_2_TiC_2_T_*x*_ MXene coated pristine and cycled MXene coated C paper showed the peaks majorly appearing from the adsorbed oxygen, at 532.1 and 533.3 eV from –C=O and –C–O, respectively. However, in MXene-coated pristine C paper peak at 530.3 eV witnesses the presence of a metal–O bond which could be Mo–O or Ti–O from the Mo_2_TiC_2_T_*x*_. There is a decreased feature of M–O in the cycled MXene coated sample (top brown plot in Fig. 6c), it also possess an intense –C=O double bonds feature which could be an indication that the oxygen atoms/groups get oxidized throughout cycling resulting in some passivating species appearing at the interface causing diminished intensity of M–O at 530.3 eV. Additionally, the broadening around 530 eV indicates further oxidation of the molybdenum and/or titanium which is to be expected since the MXene-coated samples were cycled in an aqueous environment.

Furthermore, from Fig. 6d and e, the peak for the Ti 2p (Ti–C at 455.6 eV, and Ti–O at 458.9 eV) and Ti 3p (Ti^+3^ at 37 eV and Ti^+4^ at 39.2 eV), respectively are visible, although after cycling Ti 2p features were seen to vanish probably because of formation of different type of parasitic interfacial species. This suggests that after cycling, the MXene coating could still be present in the bulk of the electrode but not at the surface of the electrode. Finally, Fig. 6f shows the Mo 3d XPS spectra for Mo_2_TiC_2_T_*x*_ MXene coated C paper, the presence of 3 main peaks at 229.7, 232.9 and 236.0 eV are overlapping in agreement with the previous reports,^35^ which confirms the characteristic peaks of the Mo–C 3d (229.7 and 232.9 eV correspond to the electrons in the 3d_5/2_ and 3d_3/2_ levels, respectively) and Mo–O 3d orbitals can still be observed at 233.15 eV and 236.28 eV (correspond to 3d_5/2_ and 3d_3/2_ level electrons, respectively) for both the used MXene coated electrode and the fresh MXene coated electrode, see Fig. 6f. The Mo–C feature at 229.7 eV in the used C paper, however, is lost after cycling (Fig. 6f, top-most plot), suggesting two possibilities: (i) a surface formation that might differ from the bulk of the electrodes, or (ii) the Mo–C bond might be oxidized during the aqueous medium cycling processes. Based on the XPS analysis, the results reveal that the interfacial parasitic layer formed after cycling, confirming the stability of the MXene coating on the untreated carbon paper electrodes.

## Conclusions

For the first time, a double transition metal MXene, molybdenum titanium carbide (Mo_2_TiC_2_T_*x*_), was successfully drop-casted on carbon papers and tested in the VRFB system. The electrochemical performance of the Mo_2_TiC_2_T_*x*_ MXene-coated carbon paper was competitive with that of heat-treated ones. The cyclic voltammetry tests revealed that the 0.1 mg cm^−2^ coating density is suitable for MXene coating. The competitive diffusion coefficient and electrochemical reaction rate coefficients of 5.51 × 10^−5^ cm^2^ s^−1^ and 7.76 × 10^−4^ cm s^−1^ were achieved. Furthermore, the VRFB performance using Mo_2_TiC_2_T_*x*_ MXene-coated carbon papers showed stable energy efficiency and excellent capacity retention for over 150 cycles. The post-analysis of the Mo_2_TiC_2_T_*x*_ MXene-coated carbon papers after VRFB tests was performed *via* XPS, and it showed that the primary elements remained on the carbon papers.

This work further broadens the horizons for the application of MXenes in VRFBs. The performance of the MXene-coated electrodes in this work implies that the simple drop-casting method is a viable alternative to energy-consuming heat treatment.

## Data availability

The original data of the study are included in the article and ESI.† Further inquiries can be directed to the corresponding authors.

## Author contributions

Emil Botling: conceptualization, methodology, validation, investigation, writing – original draft, writing – review & editing. Ritambhara Gond: writing – review & editing, investigation. Anupma Thakur: validation, writing – review & editing, advising. Babak Anasori: writing – review & editing, advising. Amirreza Khataee: conceptualization, writing – review & editing, advising.

## Conflicts of interest

The authors declare that they have no known competing financial interests or personal relationships that could have appeared to influence the work reported in this paper.

## Supplementary Material

RA-015-D5RA01163A-s001
