# Supplementary material for: Molybdenum titanium carbide (Mo2TiC2Tx) MXene coated carbon electrodes for vanadium redox flow batteries
Source: RSC Adv. 2025 Apr 29;15(17):13744–52. doi: 10.1039/d5ra01163a (PMC12038686; doi:10.1039/d5ra01163a)
Supplement: RA-015-D5RA01163A-s001 [file RA-015-D5RA01163A-s001.pdf]

Supplementary Information (SI) for RSC Advances.
This journal is © The Royal Society of Chemistry 2025

Supporting Information

Molybdenum Titanium Carbide (Mo_2TiC_2) MXene coated electrodes for Vanadium Redox Flow batteries

Emil Botling [a], Ritambhara Gond [b], Anupma Thakur [c], Babak Anasori [c,d] and Amirreza Khataee [a]

a Division of Applied Electrochemistry, Department of Chemical Engineering, KTH Royal Institute of Technology, SE-100 44, Stockholm, Sweden
b Department of Chemistry – Ångström Laboratory Uppsala University, Box 538, 751 21 Uppsala, Sweden
c School of Materials Engineering, Purdue University, West Lafayette, IN, 47907 USA
d School of Mechanical Engineering, Purdue University, West Lafayette, IN 47907 USA

Corresponding author:

khat@kth.se

Table S1. Basic properties of the carbon papers

**Carbon paper**	**Thickness** (μm)	**Porosity**	**Resistivity, through plane** ($m\Omega/cm^2$)	**Microporous layer**	**PTFE treatment**
Sigracet 28AA	190	82%	4	No	No
Toray 060	190	78%	5.8	No	5 wt%
Freudenberg H23	210	80%	4.5	No	No

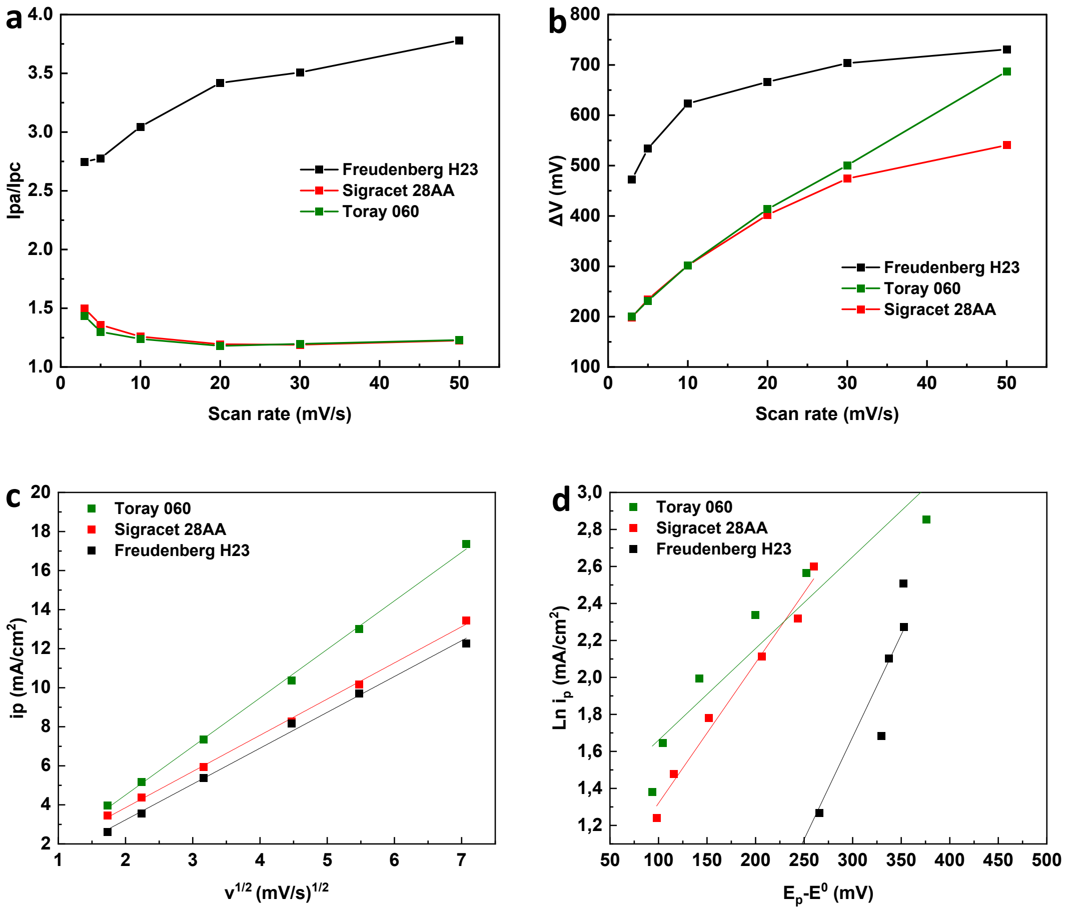

Figure S1: a) The peak-to-peak ratio Ipa/Ipc against the scan rate of the cyclic voltammetry measurement, b) The value of ΔV against the scan rate of the cyclic voltammetry measurement, c) The relationship between peak current density and the square root of the scan rate, d) The linear relationship between the natural logarithm of peak current density and peak separation.

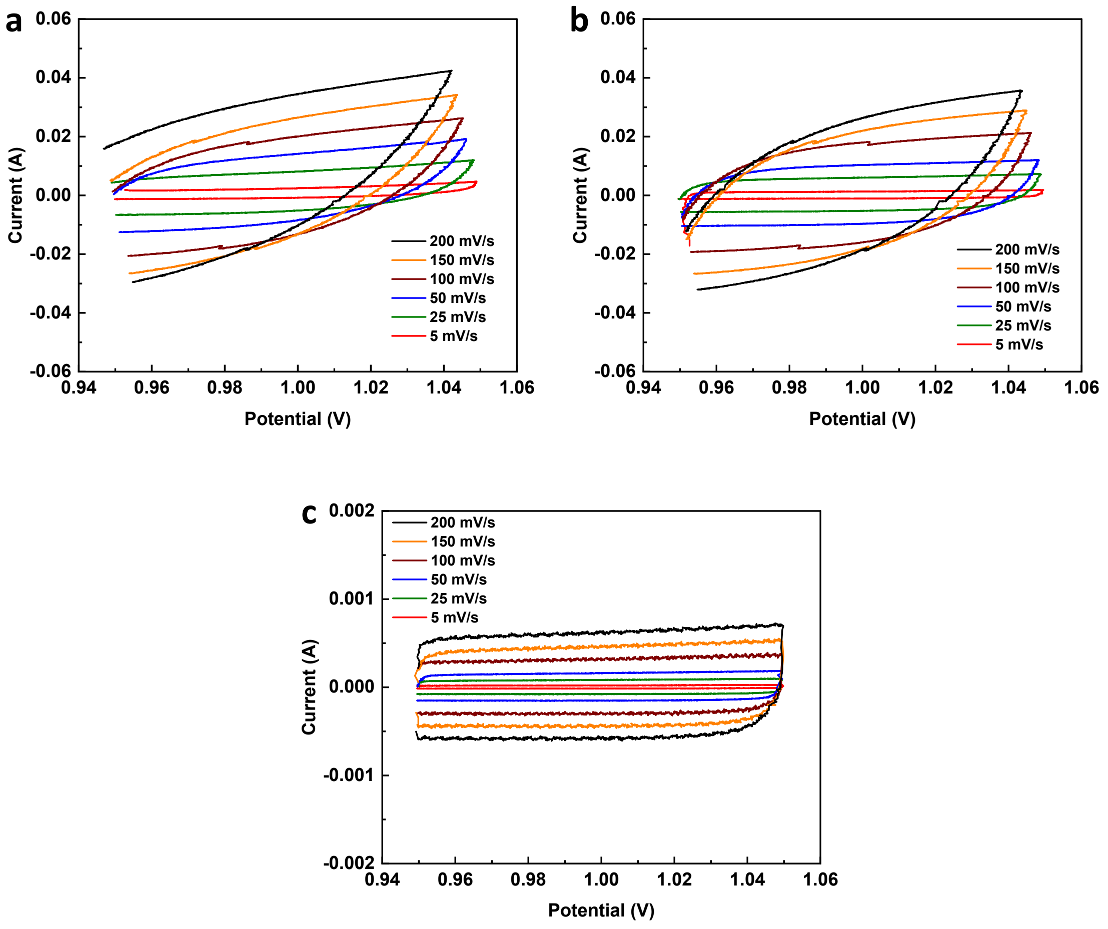

Figure S2: The cyclic voltammetry scans for the double-layer capacitance between 0,95 V and 1,05 V at different scan rates; a) Sigracet 28AA, b) Toray 060 , c) Freudenberg H23.

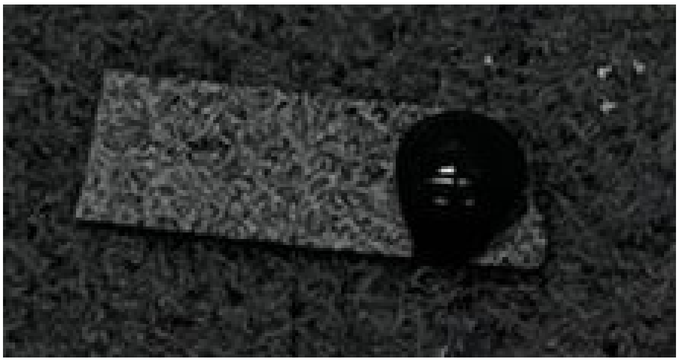

Figure S3: Unsuccessful MXene coating on Toray 060.

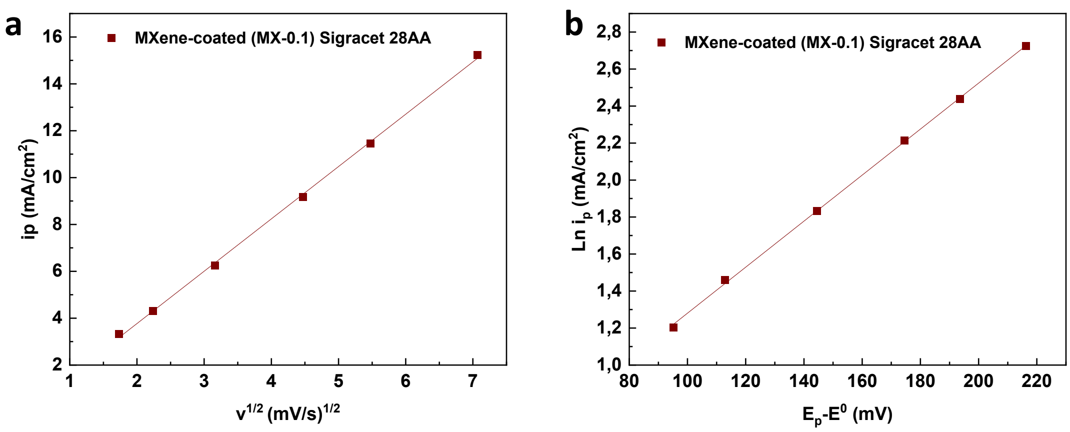

Figure S4: a) The relationship between peak current density and the square root of the scan rate, b) The linear relationship between the natural logarithm of peak current density and peak separation.

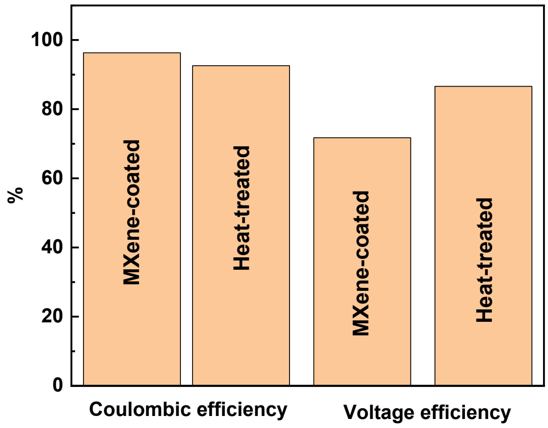

Figure S5: VRFB performance comparison using heat-treated and Mo_2TiC_2 MXene coated Sigracet 28AA on the positive side (heat-treated Sigracet 28AA was used on the negative side for both configurations); Coulombic and voltage efficiency for 150 cycles. The VRFB tests were conducted at a flow rate of 40 mL/min with 13 mL of the electrolyte on each side and was charged/discharged at constant current 100 mA/cm^2 between 0.8-1.7 V. Two layers of electrodes were stacked on each side for the configurations.
